# Comparative Dietetic Value of Fermented and Aerated Bread

**Published:** 1860-07

**Authors:** J. Dauglish

**Affiliations:** Tunbridge Wells.


### ARTICLE XXIII.

*Comparative Dietetic Value of Fermented and Aerated Bread.* By J. DAUGLISH, M. D., of Tunbridge Wells.

SINCE the new process of preparing bread has been introduced—a process which effects the raising of bread wholly by mechanical means, imparting to it the most perfect vesicular structure, while it leaves the constituents of the flour wholly unchanged and uncontaminated—there has not been wanting those who doubt whether the process of fermentation, by which bread has been hitherto prepared, is not really beneficial in other respects than that of imparting the vesicular structure to it; whether, in fact, the changes which the constituents of the flour—especially the starch—undergo, are not essential to healthy digestion in the stomach.

Although I believe there are few members of the medical profession who will be prepared to maintain that fermentation is beneficial, still, as some do hold such an opinion, and have asserted likewise that starch which has not undergone the fermentive process is wholly unfit for human food, I am desirous of stating what I believe are good reasons for rejecting the process of fermentation for the new one which I have introduced.

In order to dispose of the assertion that starch requires to be prepared by the fermentive changes to render it fit for human food, it is but necessary to remark, that the proportion which the inhabitants of the earth, who thus prepare their starchy food, bear to those who do not, is quit insignificant. Indeed it would appear that the practice of fermenting the flour or meal of the cereal grains is followed chiefly by those nations who use a mixed animal and vegetable diet, while those who are wholly fed on the products of the vegetable kingdom reject the process of fermentation entirely. Thus, the millions of India and

China, who feed chiefly on rice, take it for the most part simply boiled ; and that large portion of the human race who feed on maize, prepare it in many ways, but they never ferment it. The same is true with the potato-eater of Ireland, and with the oatmeal-eater of Scotland. Nor do we find that even wheat is always subjected to fermentation ; but the peculiar physical properties of this grain appear to have tasked man's ingenuity more than any other, to devise methods of preparing from it food which shall be both palatable and digestible. In the less civilized states, a favorite mode of dressing wheat grain has been, by first roasting and then grinding it. On the borders of the Mediterranean it is prepared in the form of maccaroni and vermicelli, while in the East it is made into hard thin cakes for the more delicate, and for the hard-working and robust into thicker and more dense masses of baked flour and water. Even in our own nurseries wheaten flour is baked before it is prepared with milk for infants' food. The necessity for subjecting wheaten grain to these manipulations arises from its richness in gluten, and from the peculiar properties of that gluten. If a few wheaten grains are taken whole and thoroughly masticated, the starchy portions will be easily separated, mixed with the saliva and swallowed, whilst nearly the whole of the gluten will remain in the mouth in the form of a tough tenacious pellet, on which scarcely any impression can be made. A similar state of things will follow the mastication of flour. In this condition, the gluten is extremely indigestible, since it cannot be penetrated by the digestive solvents, and they can only act upon its small external surface ; hence the necessity to prepare food from wheat in such a manner as shall counteract this tendency to cohere and form tenacious masses. This is the object of baking the grain and the flour as before mentioned, of making it into maccaroni, and of raising it into soft spongy bread ; by which latter means the gluten assumes a form somewhat analogous to the texture of the lungs, so that an enormous surface is secured for the action of the digestive juices ; and this I believe is the sole object to be sought in the preparation of bread from wheaten flour.

Wheat is said to be the type of adult human food. It supplies, in just proportions, every element essential to the perfect nutrition of the human organism. And yet in practice we find that the food which we prepare from it, and furnish to the inhabitants of our large towns and cities, is quite incapable alone of sustaining the health and strength of any individual. This is the more remarkable, since in Scotland we find that the food prepared from the oat, a grain possessing the same elements of nutrition as wheat, though in a coarser form—furnishes almost the exclusive diet of a very large number of the hardiest and finest portion of the population.

The deficiency of wheaten bread in affording the nourishment due to the constituents of the grain, is to be attributed solely to the mode of preparing the flour, and the process followed for making that flour into porous bread. The great object sought after both by the miller and the baker, is the production of a white and a light loaf. Experience has taught the miller that the flour which makes the whitest loaf is obtained from the centre of the grain ; but that the flour which is the most economical, and contains the largest proportion of sound gluten, is that which is obtained from the external portions of the grain. But while he endeavors to secure both these portions for his flour, he takes the greatest care to avoid as much as possible, by fine dressing, &c., the mixture with them of any part of the true external coat which forms the bran, knowing that it will cause a most serious deficiency in the color of the bread after fermentation.

It is generally supposed that the dark color of brown bread—that is of bread made from whole wheaten meal—is attributable to the colored particles of the husk or outer covering of the grain. But such is not really the case. The colored particles of the bran are of themselves only capable of imparting a somewhat orange color to bread, which is shown to be the fact when whole wheaten meal is made into bread by a process where no fermentation or any chemical changes whatever are allowed to take place. Some few years since a process was invented in America for removing the outer seed coat of the wheat grain without injuring the grain itself, by which it was proposed to save that highly nutritious portion which is torn away, adhering to the bran in the ordinary process of grinding, and lost to human consumption. The invention was brought under the notice of the French Emperor, who caused some experiments to be made in one of the Government bakeries to test its value. The experiments were perfectly satisfactory so far as the making of an extra quantity of white flour was concerned, but when this flour was subjected to the ordinary process of fermentation and made into bread, much to the astonishment of the parties conducting the experiments, and of the inventor himself, the bread was brown instead of white. The consequence, of course, has been that the invention has never been brought into practical operation.

It has been estimated that as much as ten or twelve per cent. of nutritious matter is separated adhering to the bran, which is torn away in the process of grinding, and until very lately this matter has been considered by chemists to be gluten. It has, however, been shown by M. Mège Mouries to be chiefly a vegetable ferment, or metamorphic nitrogenous body, which he has named Cerealin, and another body, vegetable Caseine.

Cerealin is soluble in water, and insoluble in alcohol. It may be obtained by washing bran, as procured from the miller, with cold water, in which it dissolves, and it may be precipitated from the aqueous solution by means of alcohol; but, like pepsin, when thus precipitated it loses its activity as a solvent or ferment. In its native state or in aqueous solution, it acts as the most energetic ferment on starch, dextrine, and glucose, producing the lactic and even the butyric changes, but not the alcoholic. It acts remarkably on gluten, especially when in presence of starch, dextrine, or glucose. The gluten is slightly decomposed at first, giving ammonia, a brown matter, and another production which caused the lactic acid change to take place in the starch and glucose. The lactic acid thus produced immediately combines its activity with that of the cerealin and the gluten is rapidly reduced to solution.

The activity of cerealin is very easily destroyed by most acids, also by the presence of alum ; and while it is the most active agent known in producing the earlier changes in the constituents of the flour, it cannot produce the alcoholic, but as soon as the alcoholic is superinduced the cerealin becomes neutralized and ceases to act any longer as a solvent. The action of cerealin upon the gluten of wheat is precisely similar to that of pepsin on the fibrine of meat. Pepsin acting alone on fibrine dissolves it, but very slowly, but if lactic acid be added solution takes place very rapidly. In like manner the starch present with the gluten of wheat is acted upon by the cerealin, and produces the necessary lactic acid to assist in the solution of the gluten by cerealin.

With the knowledge thus obtained of the properties of this substance cerealin, it is not difficult to understand why the administration of bran-tea with the food of badly-nourished children, produces the remarkable results attributed to it by men both experienced and eminent in the medical profession ; and why, also, bread made from whole wheaten meal, which contains all the cerealin of the grain, should prove so beneficial in some forms of mal-assimilation, notwithstanding the presence of the peculiarly indigestible and irritating substance forming the outer covering of the grain. It will be seen that in all the methods of bread-making hitherto adopted, the peculiar solvent properties of this body, cerealin, has been sought to be neutralized simply because it destroys the white color of the bread during the earlier stages of panary fermentation. It is by thus destroying the activity of the special digestive ferment which nature has supplied for the due assimilation by the economy of the constituents of the wheaten grain, that wheaten bread is rendered incapable of affording that sustenance to the laboring man which the Scotchman obtains from his oatmeal porridge. Although the new bread has been as yet but little more than experimentally introduced to public consumption, I have already received from members of my own profession, who have recommended it in their practice, as well as from non-professional persons, accounts of the really astonishing results that have followed its use in cases of deranged digestion and assimilation. Private gentlemen have sought interviews with me to record the history of their recovery to health, after years of suffering and misery, by the simple use of the bread as a diet. Children that have been liable to convulsive attacks from an irritable condition of the alimentary canal and nervous system, have been perfectly free from them immediately the new bread was substituted for fermented bread. And cases are now numerous that have been communicated to me by medical men of position, in which certain distressing forms of dyspepsia, which had remained intractable under every kind of treatment, have yielded as if by magic almost immediately after adopting the use of the aërated bread. The delicate flavor of the new bread renders it peculiarly grateful to the stomachs of invalids and children, as well as of those whose tastes have not become vitiated by the habitual use of baker's bread, which is slightly sour, and tastes of yeast. The new bread was supplied to two wards in Guy's Hospital, in place of the ordinary bread (which is of a very fine quality, made on the premises) for two months, and in no case were there any pieces left in the wards unconsumed, while of the fermented bread large quantities of scraps are collected daily, for the consumption of which the appetites of the patients have been deficient. I am disposed to attribute the beneficial effects of the new bread to two causes. The one to the *absence* of the prejudicial matters imparted to ordinary bread by the process of fermentation, and the other to the *presence* in the bread, unchanged, of that most essential agent of digestion and assimilation, cerealin. I believe the prejudicial matters imparted to bread by fermentation to be chiefly two—acetic acid and the yeast-plant. The first is produced in large quantities, especially in hot weather, by the oxydation, by atmospheric contact, of the alcohol produced. The second is added when the baker forms his sponge, and is also rapidly propagated during the alcoholic fermentation, and cannot of course be afterwards separated from the other materials in the manner that the yeast and the other *debris* of fermentation separate themselves from wine and beer by precipitation in the process of fining. Nor is the life of the yeast plant generally destroyed in baking, because it requires to be retained at the boiling point for some time before it is thoroughly destroyed; and bread is generally withdrawn from the oven, for economical reasons, even before the centre of the loaf has reached the temperature of 212°. It is not difficult to understand how the most painful and distressing symptoms and derangements may follow the use of bread in which the yeast-plant is not thoroughly destroyed previous to ingestion, in those cases of impaired function in which the peculiar antiseptic influence of the stomachal secretions is deficient, and is incapable of preventing the development of the yeast-plant in the stomach, and the setting up of the alcoholic fermentation to derange the whole process of digestion and assimilation.—*Medical Times.*

